# Surgical vs. nonoperative treatment for acute Achilles' tendon rupture: a meta-analysis of randomized controlled trials

**DOI:** 10.3389/fsurg.2024.1483584

**Published:** 2024-11-21

**Authors:** Lei Fan, Yunan Hu, Leng Zhou, Weili Fu

**Affiliations:** ^1^ Sports Medicine Center, Department of Orthopedic Surgery/Orthopedic Research Institute, West China Hospital, Sichuan University, Cheng Du, China; ^2^ Department of Anesthesiology, West China Hospital, Sichuan University, Cheng Du, China

**Keywords:** acute Achilles tendon rupture, conservative treatment, surgery, meta-analysis, clinic outcomes

## Abstract

**Background:**

Acute Achilles tendon rupture (AATR) is common among young individuals. There are various management options available, including conservative treatment, open surgical repair, and minimally invasive treatments. However, the optimal treatment approach remains controversial.

**Purpose:**

In this study, we conducted a thorough analysis of the existing literature to compare the clinical outcomes of surgical and nonoperative treatments for patients with AATR by conducting a meta-analysis of randomized controlled trials.

**Study design:**

Meta analysis; Level of evidence, 1.

**Methods:**

Eligible trials randomly assigned adults with AATR to surgical or conservative treatment and assessed by three independent reviewers. We searched in PubMed, Embase, and The Cochrane Library. The assessment of risk of bias was conducted by entering the data from each included study into the Revman computer program. Extracted data were meta-analyzed. Heterogeneity was evaluated using the I2 test. Pooled results were expressed as odds ratios, risk ratios (OR), and mean differences (MD).

**Results:**

The meta-analysis included a total of 14 studies and 1,399 patients, with 696 patients receiving surgical intervention and 703 patients undergoing non-surgical treatment. The follow- up duration ranged from 12 to 30 months. The surgical group was found to have a significantly lower re-rupture rate (OR: 0.30, 95% CI: 0.18–0.54; *P* < 0.00001), but also had a higher risk of other complications (OR: 3.28, 95% CI: 1.56–6.93, *P* = 0.002). The surgical group also had significantly abnormal calf (OR: 0.45, 95% CI: 0.26–0.76, *P* = 0.03). There was no statistically significant difference between the two groups in terms of returning to sports, ATRS, abnormal motion of foot and ankle, unable heel-rise, and torque for plantar flexion.

**Conclusion:**

The meta-analysis results indicate that surgical intervention for AATR is associated with a lower re-rupture rate, but a higher risk of other complications. Our assessment of life-quality and functional outcomes also suggests that surgery leads to significantly better outcomes in terms of sick leave, abnormal calf, and torque for plantar flexion. Based on these findings, we recommend that surgery is a preferable option for patients who have a higher risk of re-rupture and require a quick rehabilitation.

## Introduction

The Achilles tendon is composed of the tendinous portion of the gastrocnemius and soleus muscles, and is the strongest and largest tendon in the body. AATR is one of the most common musculoskeletal injuries, with an annual incidence ranging from 5 to 50 events per 100,000 persons (1–3). This injury can lead to significant disability and impairments in daily activities. AATR typically occurs during sports such as tennis, basketball, soccer, and badminton, although it can also occur with sudden dorsiflexion of the foot, which is a rare event (4). Diagnosis is usually made based on a thorough case history and physical examination, which may reveal a palpable gap and positive Thompson test. If the physical examination is inconclusive, clinicians may use ultrasonography or magnetic resonance imaging (MRI) to confirm the diagnosis (5, 6).

Treatment options for AATR include surgical and non-surgical management. Surgical management can include open repair, minimally invasive repair, or percutaneous repair. Non-surgical management can include the use of a cast, cast-boot, or splint with the foot placed in plantar flexion, with or without early physiotherapy (7). However, there is ongoing debate among healthcare professionals regarding the best treatment approach for AATR. While surgical management has been shown to have a lower risk of re-rupture, it also carries a higher risk of complications. On the other hand, non-surgical management, has a lower risk of complications but may have a higher risk of re-rupture (8). Cetti et al. and Möller et al. conducted studies that showed that surgical treatment of acute Achilles tendon rupture resulted in less calf muscle atrophy, better ankle movement, and a higher rate of return to sports compared to non-surgical treatment (9, 10). These findings suggest that surgery may be a more effective option for patients. However several studies (11–13) suggest that there is no significant difference in the risk of re-rupture or final functional outcome between surgical and non-surgical management of AATR.

While the previous meta-analyses have provided valuable information regarding complications and re-rupture rates, there has been less emphasis on assessing patient outcomes of life-quality and functional status after treatment (14–16). It is important to continue to gather and analyze data on the effectiveness of both surgical and non-surgical treatments for AATR, including not just complications and re-rupture rates, but also patient-reported outcomes such as quality of life and functional outcomes. Meanwhile a few of high-quality RCT-literature was published newly. Therefore the evidence needs to be re-examined by taking the new trials into consideration.

## Methods

We adhered to the 2020 PRISMA (Preferred Reporting Items for Systematic reviews and Meta-Analyses) statement in conducting this study and reporting the results (17).

### Information sources and search strategy

Three reviewers independently searched medical databases (PubMed, Embase, and The Cochrane Library) for all English-language studies published before December 2022, using the search strings, “(achilles tendon rupture) and (therapy or treatment)”.

### Eligibility and selection process

We conducted a thorough search for relevant literature, including randomized controlled trials and controlled clinical trials. We included only English-language publications and required a follow-up of at least 12 month to address long-term prognosis. We excluded case reports, case series studies, cross-sectional studies, and quasi-randomized trials and RCTs that compared different surgical methods, different conservative approaches, other non-relevant comparisons, the same trials with useless information, non-randomized treatment methods, or improper information or data. Surgical treatments included open or minimally invasive techniques, while nonsurgical management involved casting or functional bracing. We excluded non-RCT and duplicates through the use of web filters. We then manually reviewed the remaining articles based on their titles and abstracts to determine the left papers that read in full, and finally got the included papers.

### Data collection process and data items (outcomes)

Using a prepared extraction sheet, three independent reviewers extracted the following data: (1) publication information, including first author name and year of publication; (2) designation information: length of follow-up, and loss to follow-up; (3) participant information, including mean age, sex (male, n), injured side; (4) intervention information: surgical techniques including Bunnell's type suture, Krackow type suture, Kessler type suture and their modified techniques. (5) Outcomes: The main outcomes explained below were the complications, quality of life outcomes, and functional outcomes.

#### Outcomes of complications

Re-rupture means the diagnosis of rupture of achilles tendon of the injured side established after treatment. Excluding re-rupture, other complications include delayed healing, deep vein thrombosis (DVT), superficial and deep wound infections, sural nerve lesions, chronic pain, scar/skin adhesion, and wound dehiscence.

#### Outcomes of returning to sports

Back to sports was described as participating in sports after injury, including changes in sport, decreased exercise level, and pre-injury activity level.

#### Outcomes of function

Achilles tendon total rupture score (ATRS) consists of 10 items reflecting symptoms and physical activity and is currently the only validated PROM specifically for use in Achilles tendon rupture management (18). Abnormal calf include atrophy, circumference, deficits to the healthy calf, weakness, and fatty degeneration of the calf. Abnormal motion of the foot and ankle, include patient-reported outcomes (PRO) and range of motion differences greater than 5 degrees between ankles. Unable heel-rise refer to inability to perform a heel-rise, a endurance test showed in functional assessment. Plantar flexion refer to the rotational force that is generated by the muscles around ankle and strength measured as mean peak torque supine 30 degrees/s in concentric.

### Study risk of bias assessment

In order to assess the potential risk of bias for each study, we utilized the Cochrane collaboration tool, which is a recognized method for assessing bias (19). Paired reviewers evaluated at the study and outcome levels. The assessment of risk of bias was conducted by entering the data from each included study into the Revman computer program. The Cochrane risk of bias tool consists of seven items, including “Random sequence generation”, “Allocation concealment”, “Blinding of participants and personnel”, “Incomplete outcome data”, "Selective reporting”, and “Other bias”. For each item, we rated the risk of bias as low, unclear, or high.

### Effect measures and synthesis

The statistical analysis of all extracted data was carried out using Review Manager software, version 5.4. (Cochrane Collaboration). Dichotomous variables, such as the re-rupture rate, incidence of other complications, return to sports, abnormal calf, abnormal motion of foot and ankle, and unable heel-rise, were expressed as the odds ratio. Continuous variables were extracted and analyzed as the mean and standard deviation (SD), including sick leave, ATRS, and torque for plantar, and reported as the mean difference. Heterogeneity across the combined data was assessed using the *i*^2^ test. A *P*-value of less than 0.15 on the *i*^2^ test was considered an indicator of significant heterogeneity. An *i*^2^ value of less than 50% was considered homogeneous data. If the *i*^2^ value was greater than 50%, it means heterogeneity was significant. The random effects model was applied when heterogeneity was significant other than using the fixed effects model, justifying pooling. Differences were considered significant if the *P*-value was less than 0.05 and the confidence interval (CI) was 95%.

## Results

### Study selection and study characteristics

**Figure 1:**
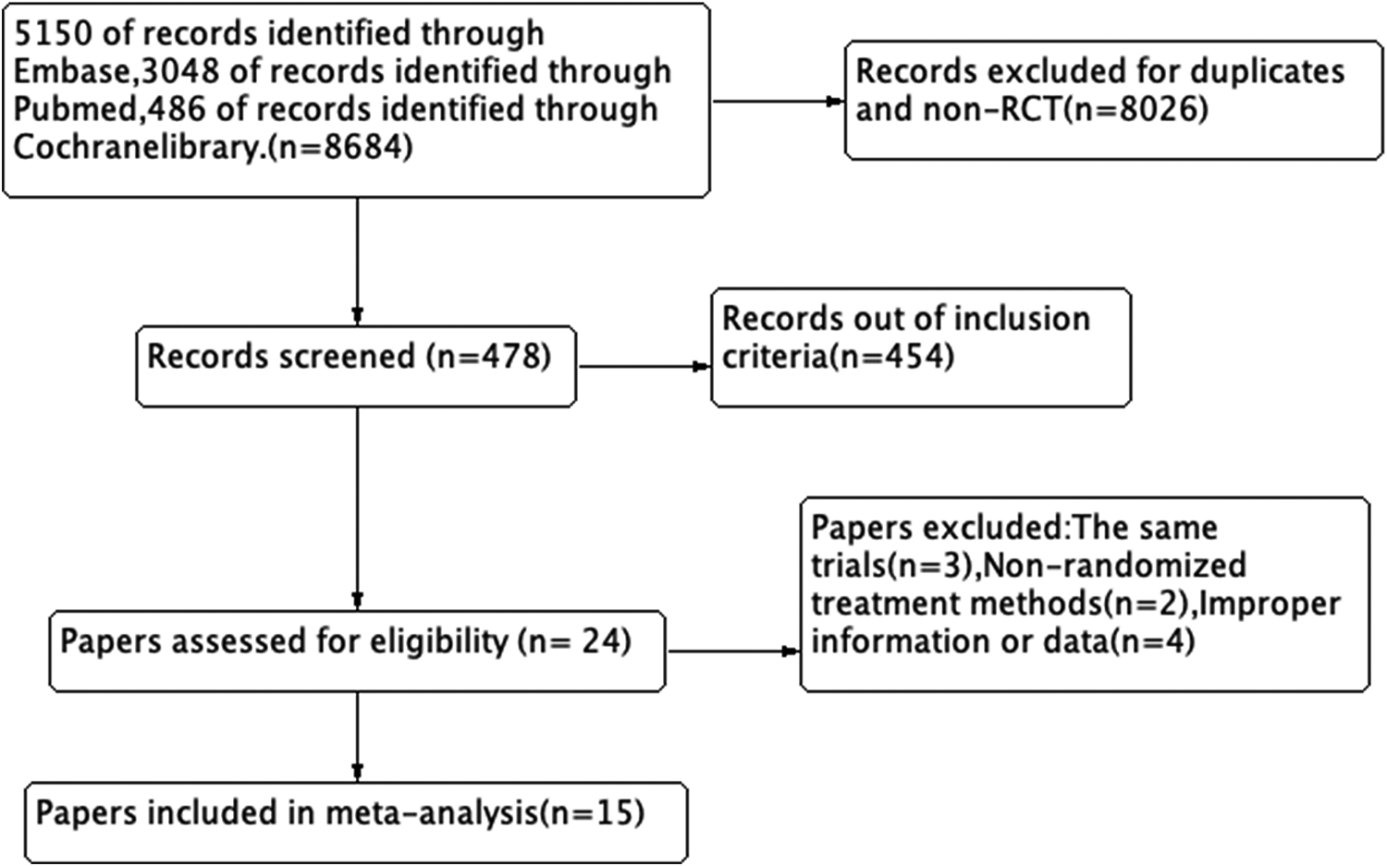
Flow diagram.

We got 8,684 records in total, and 8,026 records were excluded for duplicates or non-RCT. After screening the tittle and abstract, 454 records were excluded and 24 records were left. Finally, we reviewed the remaining articles and excluded 9 papers due to the same trials with useless information, uncorrelated randomization (non-randomized treatment methods) or improper information or data. We produced a PRISMA flowchart based on our search results and inclusion/exclusion criteria (Figure 1).

A total of 14 studies (9–13, 20–29) were included, and the most recent paper was published in 2022. In total, 1,399 patients were analyzed, with 696 receiving surgical intervention and 703 receiving non-surgical treatment. Of the patients included, 257 (18.4%) were female and 1,142 (81.6%) were male. The most recent paper included in the review was published in 2022. The mean age of patients in the surgical group ranged from 37.2 to 42 years old in each studies, while the mean age of patients in the conservative group ranged from 37.8 to 45.2 years old. Additionally, the left Achilles tendon was more commonly ruptured than the right, based on data from 10 studies that mentioned the injured side. Out of 1,088 total cases mentioned in these studies, 570 (52.4%) were on the left side and 518 (47.6%) were on the right side. Three main surgical techniques and their modified techniques were used, including Bunnell's type suture, Krackow type suture, and Kessler type suture. However, specific techniques were not mentioned in three studies (20, 23, 26). The follow-up duration ranged from 12 to 30 months, with a minimum follow-up duration of 12 months. The effective follow-up rate ranged from 73% to 100%. The characteristics of included study and results of individual studies were shown in Table 1.

**Table 1:** Characteristics of included RCTs.

Study	Patient	Female/male	Mean age	Right/left	Operation method	Follow-up	outcome
Cetti et al. (9)	Sur 56 Non 55	Sur 9/47 Non 10/45	Sur 37.2 Non 37.8	Sur 21/35 Non 25/30	Bunnell's end-to-end method,	12 Mon	Patients’ comments on daily discomfort. sick leave time, resumption of sports activities. Ankle movements (goniometer measurements), calf circumferences. Complications.
Fischer et al. (20)	Sur 23 Non 22	Sur 4/26 Non 3/27	Sur 39.6 Non 45.2	Sur 15/15 Non 17/13	Conventional open suture	24 Mon	Gait, motion of the foot and ankle (plantar flexion and dorsiflexion force), occurrence of complications, examination of the local wound and soft tissues, AOFAS-AH of the VAS FA and the SF-36 questionnaire, hop test.
Heikkinen et al. (21)	Sur 30 Non 25	Sur 2/30 Non 2/25	Sur 40 Non 39	UK	repaired with the Krackow technique	18 Mon	Volume of calf muscles, fatty degeneration of muscles and Achilles tendon length, calf muscle isokinetic strength, ankle peak isokinetic torque, Angle specific peak torque at 0°,10°, and 20° of plantar flexion.
Keating and Will (22)	Sur 37 Non 39	Sur 11/28 Non 9/32	Sur 41.2 Non 39.5	UK	End to end, Kessler stitch	12 Mon	Muscle dynamometry, rate of rerupture. Any other complications, Short Musculoskeletal Function Assessment (Functional outcome), return to work, to sport, range of plantar and dorsiflexion (goniometer).
Lantto et al. (23)	Sur 29 Non 28	Sur 2/30 Non 3/25	Sur 40 Non 39	UK	end-to-end open repair	18 Mon	Leppilahti Achilles tendon performance score, isokinetic calf muscle strength, and RAND 36Item Health Survey Re-rupture, wound infection.
Manent et al. (24)	Sur 12 Non 11	Sur 1/11 Non 1/10	Sur 42 Non 40.5	Sur 2/9 Non 4/8	double Bunnel suture	12 Mon	Scored pain intensity, Standing heelrise, Return to sports: ATRS, VISA, AOFAS Muscular strength, Calf circumference, Plantarflexion, Patients’ global impression Complications.
Metz et al. (11)	Sur 42 Non 41	Sur 11/31 Non 6/35	Sur 40 Non 41	Sur 14/28 Non 20/21	Bunnel-type suture	12 Mon	Time to work resumption, return to sports after rupture, VAS for satisfaction and pain, Leppilahti score.
Möller et al. (10)	Sur 59 Non 53	Sur 8/51 Non 5/48	Sur 39.6 Non38.5	Sur 25/34 Non 23/30	End to end, modified Kessler	24 Mon	Dorsiflexion, plantarflexion, calf circumference, isokinetic strength, heel-raise test, VAS for subjective results of treatment, FIL, satisfaction, time to return to work Re-rupture, wound infection, nerve injury, DVT.
Myhrvold et al. (26)	Sur 176 Non 178	Sur 44/132 Non42/136	Sur 39.9 Non 39.9	Sur 83/93 Non 91/87	Open repair	12 Mon	Achilles’ tendon Total Rupture Score (ATRS), physical functioning, (SF-36), incidence of tendon rerupture, physical performance.
Nilsson-Helander et al. (12)	Sur 49 Non 48	Sur 9/40 Non 9/39	Sur 40.9 Non 41.2	Sur 23/26 Non 27/21	End to end, modified Kessler	24 Mon	ATRS, PAS, jump test, strength test, muscular endurance test Re-rupture, wound infection, nerve injury. Functional Test Performance Score.
Nistor (27)	Sur 46 Non 61	Overall 11/96	Overall 41	Overall 48/58	sutured with the Bunnell type,	30 Mon	Measurements of range of motion, calf circumference, tendon width, and strength of plantar flexion. The ability to walk and to stand on tiptoe also was recorded.
Olsson et al. (28)	Sur 43 Non 45	Sur 10/39 Non 4/47	Sur 39.8 Non 39.5	Sur 25/24 Non 35/16	End to end, modified Kessler	12 Mon	ATRS, PAS, FAOS, EQ-5D, jump test, strength test, muscular endurance test Re-rupture, wound infection, DVT, nerve injury.
Twaddle and Poon (29)	Sur 20 Non 22	Sur 6/14 Non 8/14	Sur 41.8 Non 40.3	Sur 10/10 Non 10/12	End to end, Krackow stitch	12 Mon	MFAI, Dorsiflexion, Plantarflexion, calf circumference, Re-rupture.
Willits et al. (13)	Sur 72 Non 72	Sur 13/59 Non 13/59	Sur 39.7 Non 41.1	UK	End to end, Krackow stitch	24 Mon	Leppilahti score, range of motion and isokinetic strength (dorsiflexion, plantarflexion), calf circumference.

Sur, surgical group; Non, nonoperation group; ATRS, achilles tendon total rupture score; Mon, month; AOFAS-AH, American orthopaedic foot and ankle society ankle-hindfoot score; The SF-36, assesses the general health-related quality of life, UK, unknown; EQ-5D, EuroQol group questionnaire; FAOS, foot and ankle outcome score; FIL, functional index for the leg and ankle; MFAI, musculoskeletal functional assessment index; PAS, physical activity scale; ROM, range of motion; SMFA, short musculoskeletal function assessment; VAS, visual analog scale.

### Risk of bias in studies

**Figure 2:**
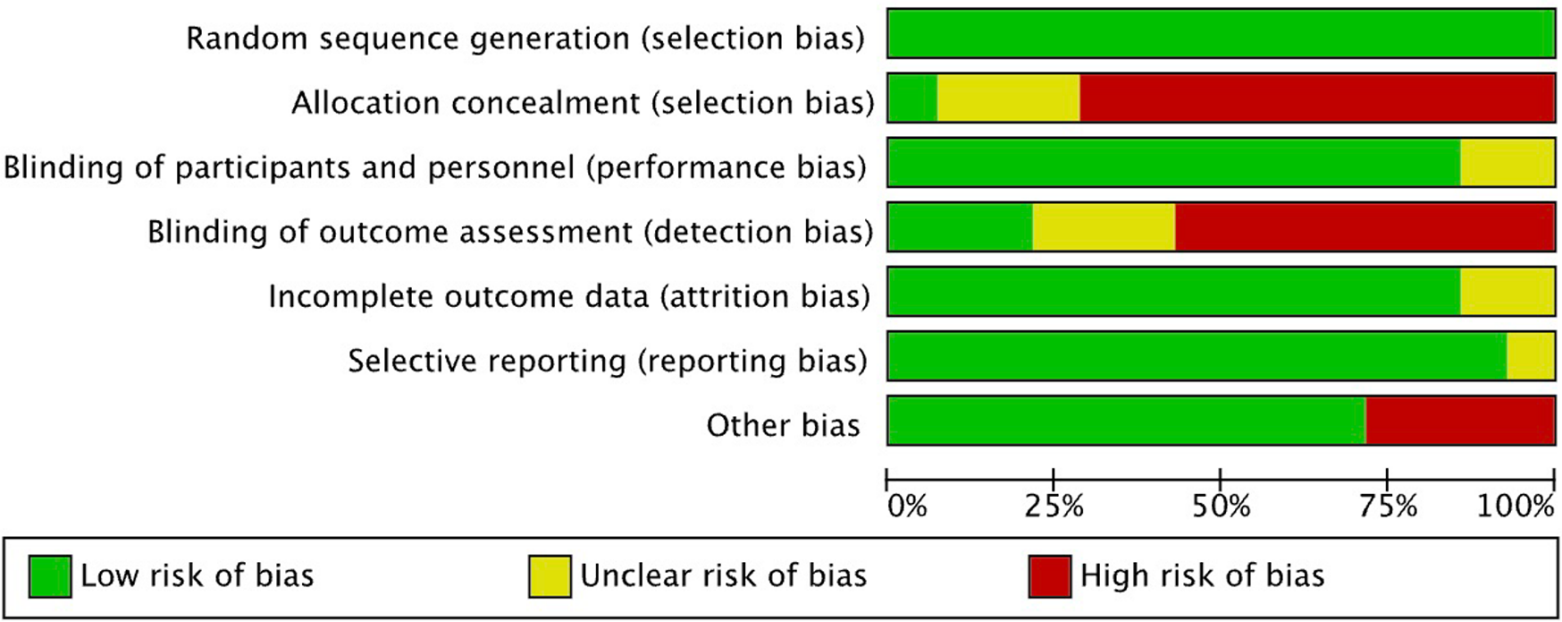
Risk of bias graph.

**Figure 3:**
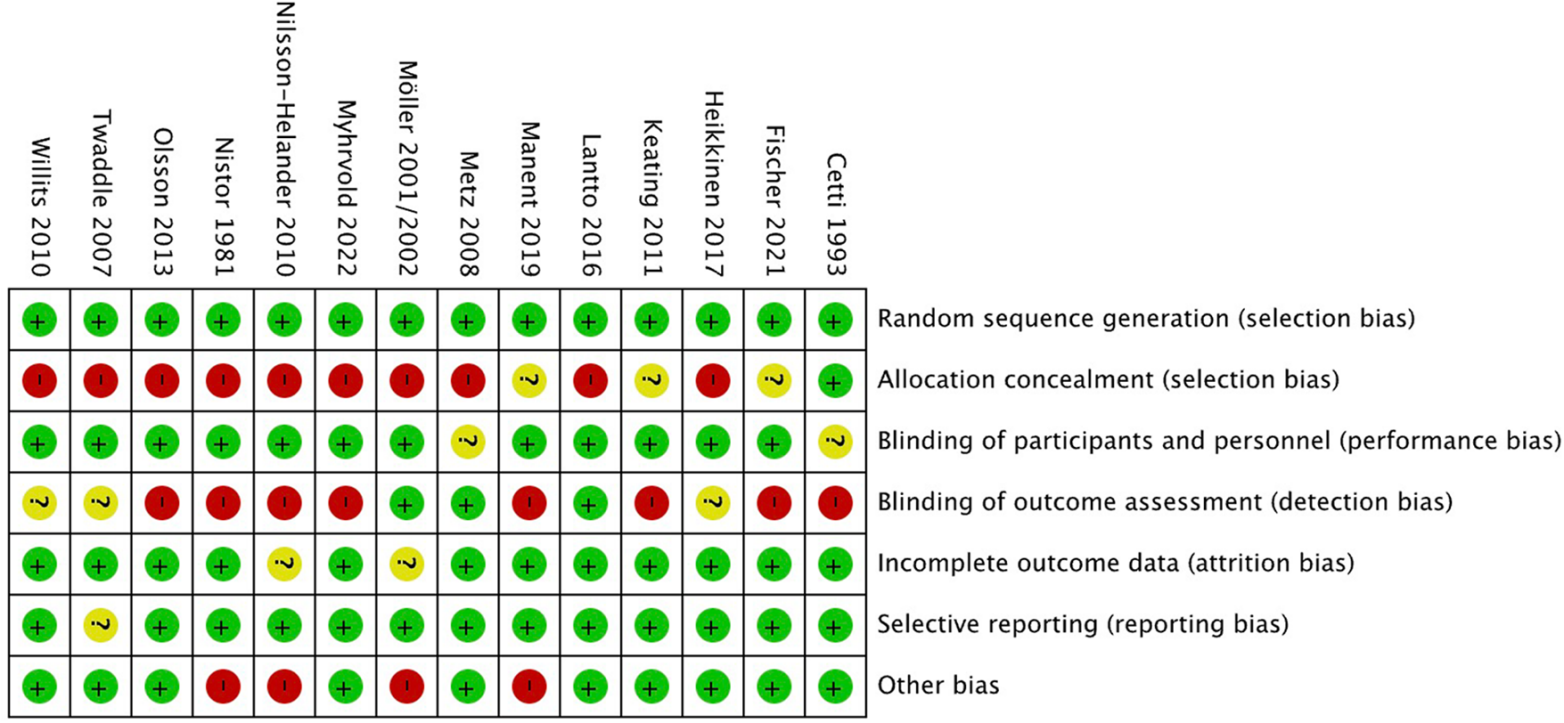
Risk of bias summary.

The inconsistent reporting of randomization or concealment methods lead to a high selection bias. The patients were not blinded to the allocated treatment, their choice may influenced by the cost, age, risk of surgery, and patient's knowledge. The study's risk of performance bias was deemed low as the majority of the outcome measures assessed were objective and unlikely to be influenced by patient factors. However, the risk of detection bias was deemed high since many of the outcomes were evaluated by investigators with unclear or insufficient blinding. The risks of attrition bias and reporting bias were considered low, as the dropout rates were minimal and all outcome measures described in the methods section were reported. It is worth noting that some studies may have introduced bias due to inadequate evaluation of homogeneity between the treatment groups. And the results of the assessment are displayed in Figures 2, 3.

### Re-rupture and other complications

#### Re-rupture rate

**Figure 4:**
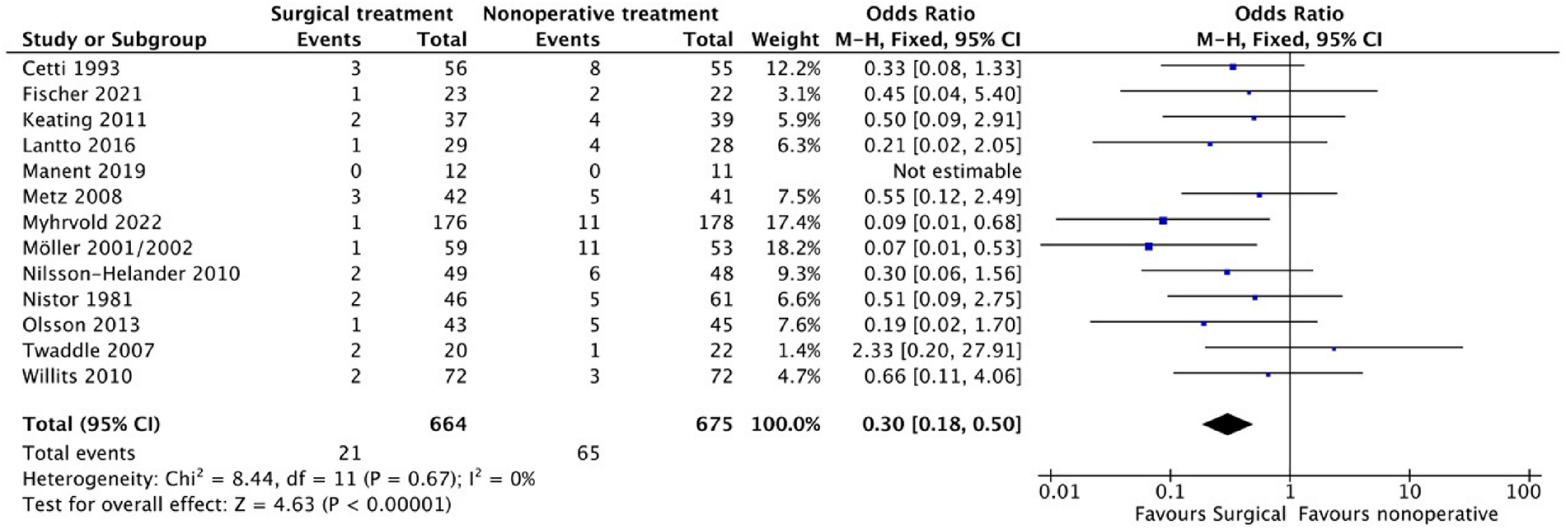
Rerupture.

Thirteen of the included studies (9–13, 20, 22–24, 26–29) reported on the re-rupture rate and complications. Out of 664 patients in the surgical group, 21 experienced re-rupture, while 65 out of 675 in the non-surgical group experienced the same. Although most literature showed a higher re-rupture rate, the difference was not significant in most studies. The pooled results from the meta-analysis (Figure 4) revealed a statistically significant lower re-rupture rate in the surgical group (OR: 0.30, 95% CI: 0.18–0.54; *P* < 0.00001), with no significant heterogeneity (*i*^2^ = 0%).

#### Other complications

**Figure 5:**
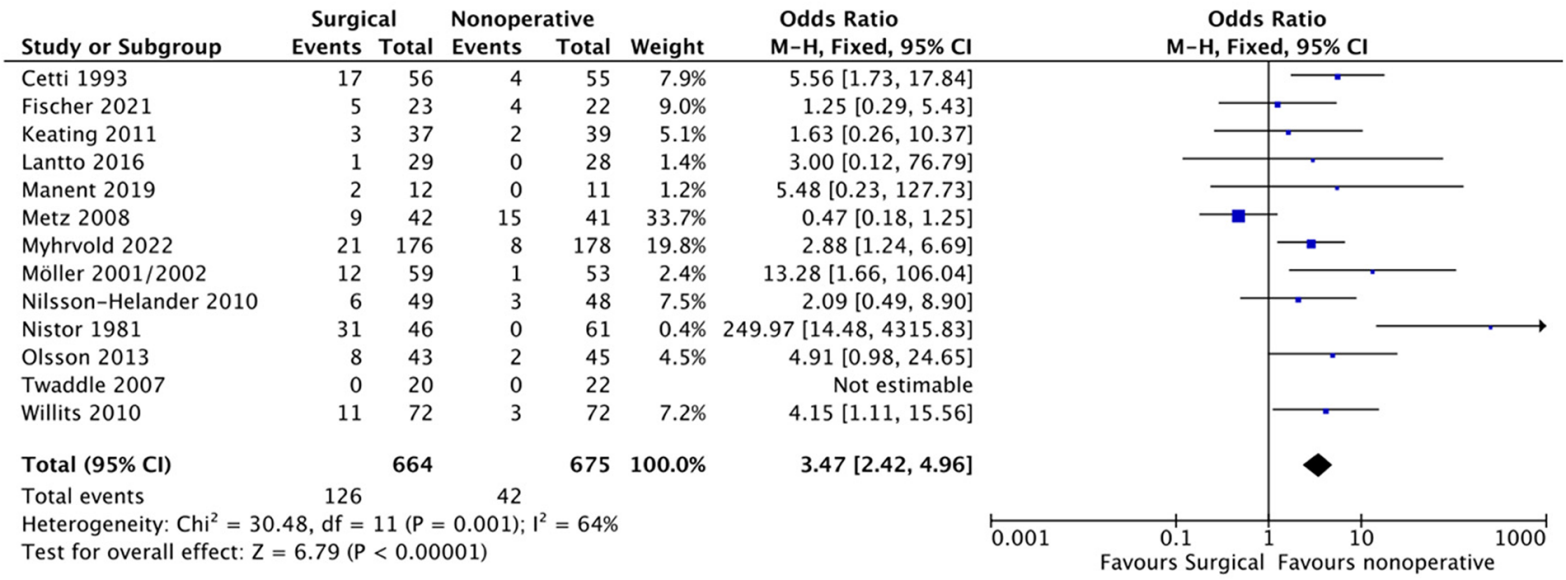
Other complication.

Of the 664 patients in the surgical group, 126 experienced the other complications, while 42 of the 675 patients in the non-surgical group experienced them. The pooled results from the meta-analysis (Figure 5) revealed a statistically significant higher rate of these complications in the surgical group (OR: 0.30, 95%) CI: 0.18–0.54; *P* = 0.002). However, significant heterogeneity (*i*^2^ = 64%) was observed, and a random effects model was applied.

#### Back to sports

**Figure 6:**
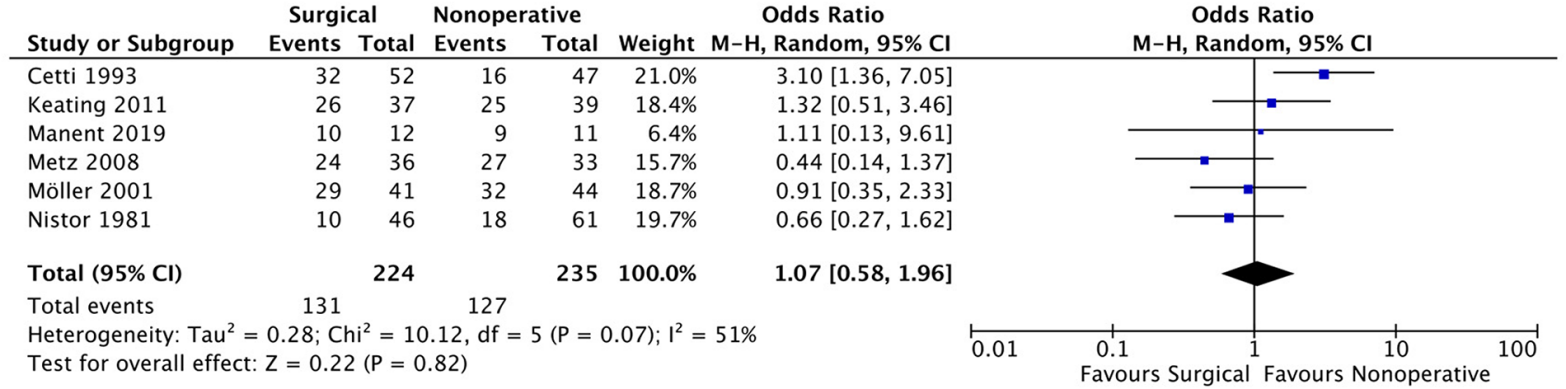
Return to sports at the same level with pre-injury.

Regarding the ability to return to sports and physical activity, six studies (9–11, 22, 24, 27), 131 patients out of 224 in the surgical group and 127 out of 235 patients in the non-surgical group were able to resume their pre-injury activity level. The meta-analysis (Figure 6) showed no significant difference between the two groups (OR: 1.07, 95% CI: 0.58–1.96; *P* = 0.82). However, high heterogeneity between the included studies required the use of a random effects model (*i*^2^ = 51%).

**Figure 7:**
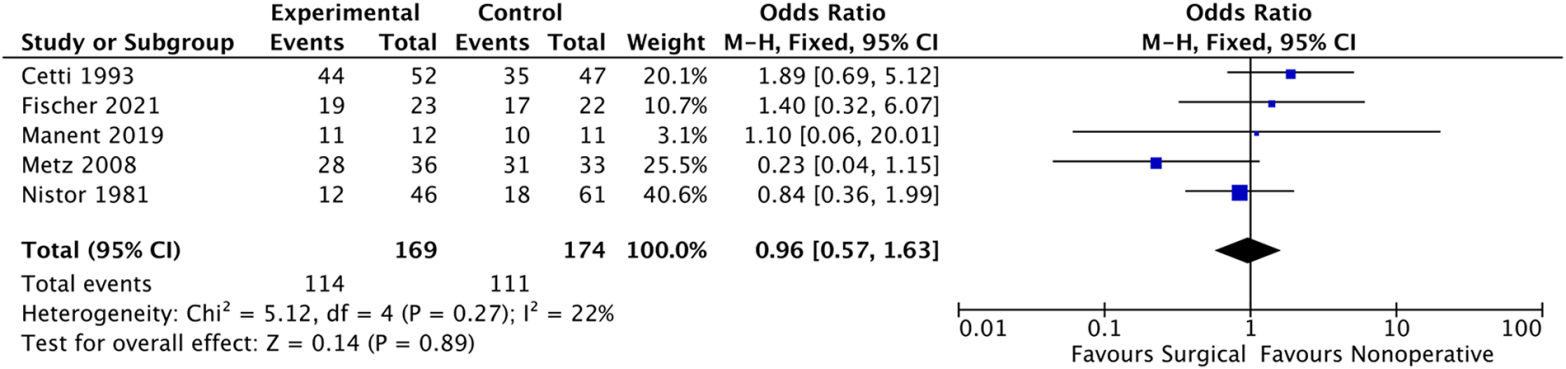
Participate in sports after treatment.

While five studies (9, 11, 20, 24, 27) reported on the return to sport, including changes in sport, decreased exercise level, and pre-injury activity level. Of the patients who underwent surgery, 114 out of 169 were able to return to sports, and 111 out of 174 patients in the non-surgical group resumed sports. The meta-analysis (Figure 7) showed no significant difference between the two groups (OR: 0.96, 95% CI: 0.57–1.63; *P* = 0.71). The fixed effect model was used due to low heterogeneity between the included studies (*i*^2^ = 22%).

**Figure 8:**
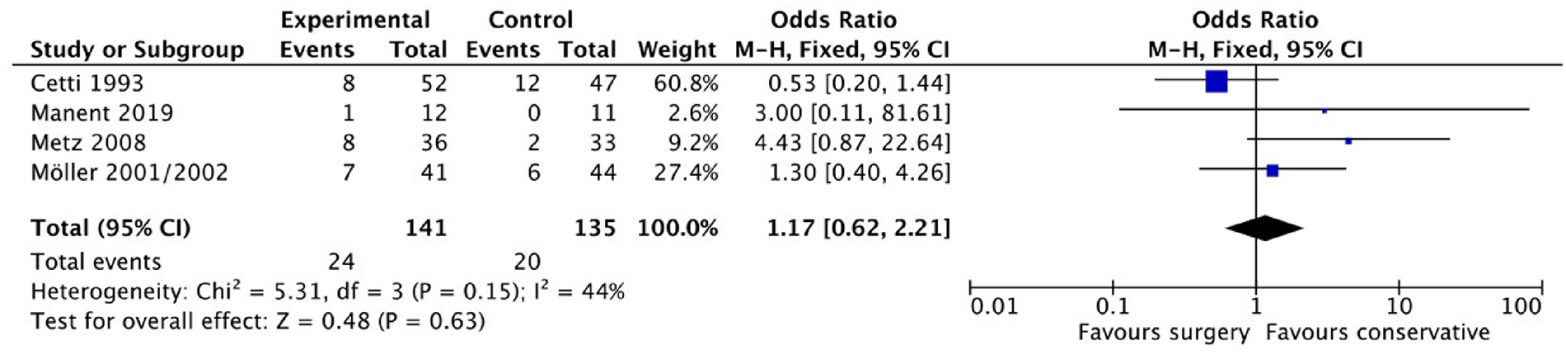
Stopped sports.

While four studies (9–11, 24) reported on the stopped sports. Of the patients who underwent surgery, 24 out of 141 terminated sports, and 20 out of 135 patients in the non-surgical group resumed sports. The meta-analysis (Figure 8) showed no significant difference between the two groups (OR: 1.17, 95% CI: 0.62–2.21; *P* = 0.15). The fixed effect model was used due to low heterogeneity between the included studies (*i*^2^ = 44%).

### Functional outcome

#### ATRS (achilles tendon total rupture score)

**Figure 9:**
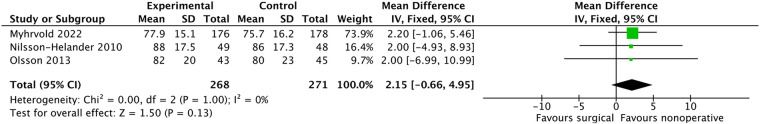
ATRS.

Based on the information provided, three studies (12, 26, 28) reported the ATRS. In total, there were 268 patients in the surgical group and 271 patients in the non-operative group. The average ATRS reported in the surgical group in the three studies were 77.9 (SD 15.1), 88 (SD 17.5), and 82 (SD 20), while the average ATRS reported in the non-operative group were 75.7 (SD 16.2), 86 (SD 17.3), and 80 (SD 23). The pooled results from the meta-analysis (Figure 9) showed no statistically significant difference between the two groups (MD (Mean difference) 2.15, 95% CI (−0.66, 4.95), *P* = 0.13). The heterogeneity between the included studies was not significant (*i*^2^ = 0%).

#### Abnormal calf

**Figure 10:**
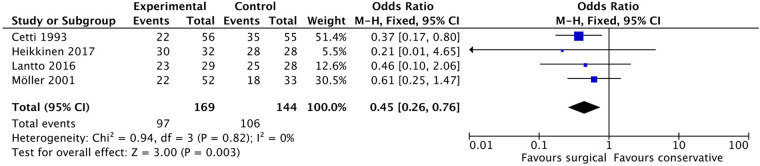
Abnormal calf.

Seven studies (9, 10, 13, 21, 23, 24, 29) reported the outcome of the abnormal calf. Four of these studies (9, 10, 21, 23, 29) were included for the complete data. In the surgical group, 97 out of 169 patients reported abnormal calf conditions, while in the non-surgical group, 106 out of 144 patients reported similar abnormalities. The pooled results from the meta-analysis (Figure 10), as shown in Figure 12, revealed a statistically significant difference between the two groups (OR: 0.45, 95% CI: (0.26–0.76, *P* = 0.03). Notably, heterogeneity between the included studies was not significant (*i*^2^ = 0%).

#### Abnormal motion of the foot and ankle

**Figure 11:**
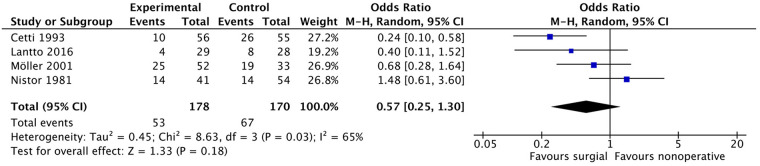
Abnormal motion of the foot and ankle.

Four studies (9, 10, 23, 27) investigated abnormal motion of the foot and ankle in patients. Among the patients, 53 out of 178 in the surgical group and 67 out of 170 in the non-surgical group reported abnormal motion. The pooled results (Figure 11) from the meta-analysis did not show a statistically significant difference between the two groups (OR: 0.57, 95% CI: 0.25–1.30, *P* = 0.18). However, heterogeneity between the included studies was significant (*i*^2^ = 65%).

#### Unable heel-rise

**Figure 12:**
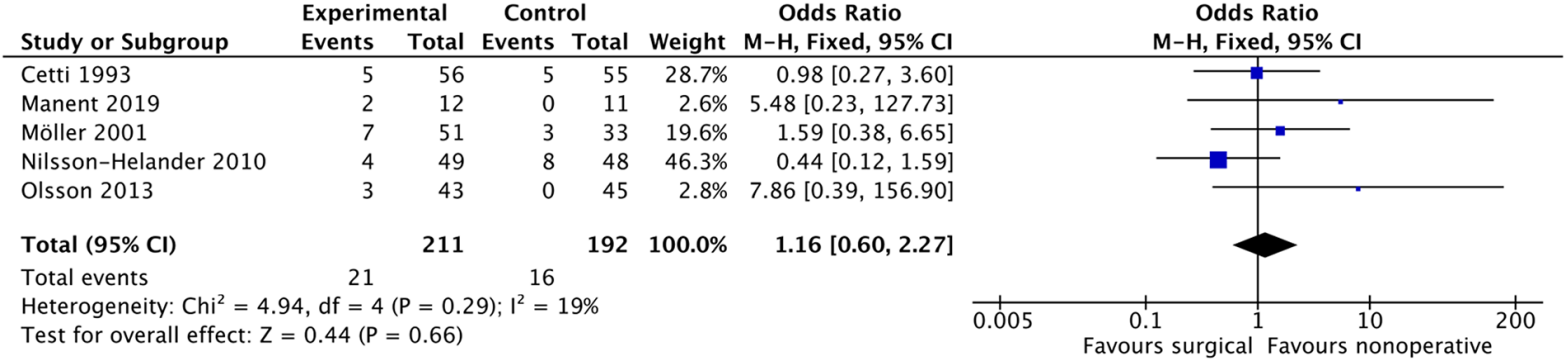
Unable heel-rise.

Five studies (9, 10, 12, 24, 28) included the unable heel-rise as a component of patient-reported outcome (PRO), endurance test, or functional assessment. Out of 210 patients in the surgical group and 192 patients in the non-surgical group, 21 and 16 patients, respectively, reported being unable to perform the heel-rise. The pooled results from the meta-analysis (Figure 12) did not demonstrate any statistically significant difference between the two groups (OR: 1.16, 95% CI: 0.60–2.27, *P* = 0.66). Heterogeneity between the included studies was not significant (*i*^2^ = 19%).

#### Torque for plantar flexion

**Figure 13:**
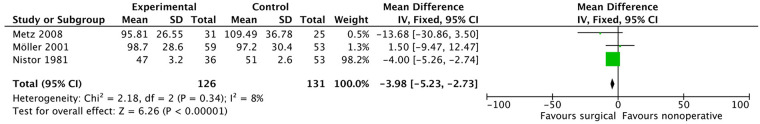
Torque for plantar flexion.

Three studies (11, 25, 27) examined torque for plantar flexion, with a total of 268 patients in the surgical group and 271 patients in the non-operative group. The average torque values for the surgical group were 95.81 N·m (SD 26.55), 98.7 N·m (SD 28.6), and 47 N·m (SD 3.2), while the corresponding values for the non-operative group were 109.49 N·m (SD 36.78), 97.2 N·m (SD 30.4), and 51 N·m (SD 2.6). The pooled results from the meta-analysis (shown in Figure 13) indicated no statistically significant difference between the two groups (MD −3.98, 95% CI: (−5.23, −2.73 *P* < 0.00001), and there was no significant heterogeneity between the included studies (*i*^2^ = 8%).

## Discussion

The ultimate goal of treating Achilles tendon rupture is to achieve optimal functional outcomes and the highest possible quality of life while minimizing complications. Our meta-analysis reveals that operative treatment is significantly associated with a lower re-rupture rate (3.16% vs. 9.62%) compared to nonoperative treatment. However, we also found that operative treatment resulted in a significantly higher rate of other complications (18.98% vs. 6.22%) which is similar to previous studies (8, 15). Complications are a significant factor that can impact a patient's life-quality and functional outcome after AATR treatment. The decision to choose surgery over non-operative management remains controversial due to the lower re-rupture rate but higher incidence of complications associated with surgery. However, it's worth noting that major complications such as wound/skin infection (30) can be mitigated through proper postoperative care and antibiotic use. Furthermore, recent studies have suggested that minimally invasive surgical techniques can help reduce the rates of complications (31, 32). Therefore, surgery may now be a more favorable option compared to the past.

To assess the life-quality of the patients, the studies used various assessment tools such as SF-36, EQ-5D, VAS, and RAND 36 Item, but finding no significant difference in long-term follow-up in these assessments. We also looked at the patients' ability to return to sports and divided them into two subgroups:those who returned to the same level as before the injury and those who still participated in sports. The forest plot showed no significant difference, but both subgroups showed a minor higher rate of returning to sports in surgical group, which is consistent with previous meta-analyses. Four studies have reported that patients often cease participating in sports after treatment for Achilles tendon injuries. Notably, in the surgical treatment group, the proportion of patients who stop exercising post-surgery is higher, though not statistically significant. This may be related to post-operative rehabilitation protocols and the psychological desire to avoid subsequent surgeries. Patients are also concerned about sick leave and want to return to work as soon as possible. Two studies found that the surgical group may allow for earlier return to work, which is similar to Brandon J. Erickson et al.'s study (33). The cost of treatment is another important factor that can influence patients' decision-making and quality of life. Few original papers were found on the cost of treatment, but recent studies showed that surgical management was more expensive compared to non-surgical management. However, the cost-effectiveness results indicate that surgical treatment is 57% likely to be cost-effective (34).

Various functional outcomes, ATRS, AOFAS-AH, FAOS, FIL, Leppilahti score, MFAI, PAS, ROM, SMFA and VAS, were used to assess the treatment of AATR, which makes it difficult to analyze a particular outcome. We extract the common components of the assessments, such as abnormal calf, abnormal motion of ankle and foot, heel-rise. The ATRS is a highly reliable, valid, and sensitive patient-reported instrument commonly used to evaluate limitations after treatment for total AATR (35, 36). The ATRS scores in the surgical group indicate better function, but the forest plot result of ATRS showed no significant difference, which is consistent with three original studies. Abnormal calf, a general concept, was reported in seven studies, with atrophy and decreased circumference as the main problems. Patients in the nonoperative group have a higher risk of abnormal calf (57.40% vs. 73.61%). However, the influence of abnormal calf on function or weakness is unclear. Soleus muscle atrophy in the affected leg could be compensated for by hypertrophy of the Flexor hallucis longus and deep flexors (21). Abnormal motion of the foot and ankle, a symptom of dysfunctional ankle, showed favorable results for the surgical group, but the difference was not significant. The heel-rise test for muscular endurance is recommended as a measure of functional recovery after AATR and has often been used for evaluation in treatment studies (37). Heel rise previously reported to favor functional outcomes regarding work and height (16), but this meta-analysis is the first to mention patients unable to heel-rise. The surgical group had a higher but not statistically significant rate of unable heel-rise, perhaps due to a higher incidence of scar/skin adhesion or other complications. The Achilles tendon serves a basic function of connecting the soleus and gastrocnemius muscles to the calcaneus bone to allow plantar flexion about the ankle joint. Torque for plantar flexion reflects ankle strength, and the forest plot result suggests surgery is a better choice, but the difference between the data is remarkable (27). Further research is needed. Eadric Bressel et al. suggest that changes in strength and peak passive torque may be chronic adaptations associated with Achilles tendon rupture (38).

Compared to previous meta-analyses, this study included a larger number of high-quality randomized control trials, comprising a total of 14 studies and 1,399 patients, which is the largest sample size included in a meta-analysis to date. The outcomes examined were more comprehensive and focused on important indicators of quality of life and function, such as return to sprots, abnormal motion of the foot and ankle, inability to perform a heel-rise, and torque for plantar flexion. However variations in surgical techniques and follow-up protocols were noted among the studies. It is worth noting that the studies included in the meta-analysis spanned over several years, and therefore, the overall quality of care and rehabilitation programs varied. This variability may have introduced some level of heterogeneity into the analysis and affected the results. When the studies (20, 24, 26) included three subgroups (open, minimally invasive, and conservative), we chose the open subgroup as the surgical group to reduce heterogeneity, as open surgery is the main surgical technique used in other studies. Another limitation of this meta-analysis is that all included studies are short-term follow-ups (12 to 30 months), so the conclusions primarily reflect short-term effects. Due to the limited follow-up period, it is not yet possible to assess the long-term impact of the intervention or treatment effects, which introduces some uncertainty in predicting long-term outcomes. Therefore, future studies with longer follow-up periods are needed to verify the sustained effectiveness and long-term safety of this intervention.

## Conclusion

The meta-analysis results indicate that surgical intervention for AATR is associated with a lower re-rupture rate, but a higher risk of other complications. Our assessment of life-quality and functional outcomes also suggests that surgery leads to significantly better outcomes in terms of abnormal calf, and torque for plantar flexion, however the sample size is limited. Other outcomes (back to sports, ATRS, abnormal motion of ankle) only show a trend towards favoring surgery, but they do not reach statistical significance. Based on these findings, we recommend that surgery is a preferable option for patients who have a higher risk of re-rupture and require a quick rehabilitation. For normal patients there is no difference between the two methods.

## Data Availability

The original contributions presented in the study are included in the article/Supplementary Material, further inquiries can be directed to the corresponding author.
